# Supplementary material for: Resolving the full spectrum of human genome variation using Linked-Reads
Source: Genome Res. 2019 Apr;29(4):635–45. doi: 10.1101/gr.234443.118 (PMC6442396; doi:10.1101/gr.234443.118)
Supplement: Supplemental Material [file supp_gr.234443.118_Supplemental_longranger-2.2.zip › longranger-2.2.tar.bz2!/lr2.2-public/lib/stan/cmdstan/stan/doc/api/html/agrad_2fwd_2functions_2lbeta_8hpp_source.html]

Stan: src/stan/agrad/fwd/functions/lbeta.hpp Source File


|  |  |
| --- | --- |
|  | Stan  2.5.0  probability, sampling & optimization |


- Main Page
- Related Pages
- Namespaces
- Classes
- Files

- File List
- File Members

All Classes Namespaces Files Functions Variables Typedefs Enumerations Enumerator Friends Macros Pages

- src
- stan
- agrad
- fwd
- functions

lbeta.hpp

Go to the documentation of this file.

1 #ifndef STAN\_\_AGRAD\_\_FWD\_\_FUNCTIONS\_\_LBETA\_HPP

2 #define STAN\_\_AGRAD\_\_FWD\_\_FUNCTIONS\_\_LBETA\_HPP

3

4 #include <stan/agrad/fwd/fvar.hpp>

5 #include <stan/meta/traits.hpp>

6 #include <boost/math/special\_functions/digamma.hpp>

7 #include <stan/math/functions/lbeta.hpp>

8

9 namespace stan {

10

11  namespace agrad {

12

13  template <typename T>

14  inline

15  fvar<T>

16  lbeta(const fvar<T>& x1, const fvar<T>& x2) {

17  using stan::math::lbeta;

18  using boost::math::digamma;

19  return fvar<T>(lbeta(x1.val\_, x2.val\_),

20  x1.d\_ \* digamma(x1.val\_)

21  + x2.d\_ \* digamma(x2.val\_)

22  - (x1.d\_ + x2.d\_) \* digamma(x1.val\_ + x2.val\_));

23  }

24

25  template <typename T>

26  inline

27  fvar<T>

28  lbeta(const double x1, const fvar<T>& x2) {

29  using stan::math::lbeta;

30  using boost::math::digamma;

31  return fvar<T>(lbeta(x1, x2.val\_),

32  x2.d\_ \* digamma(x2.val\_) - x2.d\_ \* digamma(x1 + x2.val\_));

33  }

34

35  template <typename T>

36  inline

37  fvar<T>

38  lbeta(const fvar<T>& x1, const double x2) {

39  using stan::math::lbeta;

40  using boost::math::digamma;

41  return fvar<T>(lbeta(x1.val\_, x2),

42  x1.d\_ \* digamma(x1.val\_) - x1.d\_ \* digamma(x1.val\_ + x2));

43  }

44  }

45 }

46 #endif

stan::agrad::lbeta

fvar< T > lbeta(const fvar< T > &x1, const fvar< T > &x2)

**Definition:** lbeta.hpp:16

stan::agrad::fvar

**Definition:** fvar.hpp:13

traits.hpp

stan::math::lbeta

boost::math::tools::promote\_args< T1, T2 >::type lbeta(const T1 a, const T2 b)

Return the log of the beta function applied to the specified arguments.

**Definition:** lbeta.hpp:59

fvar.hpp

stan::agrad::digamma

fvar< T > digamma(const fvar< T > &x)

**Definition:** digamma.hpp:16

stan::agrad::fvar::d\_

T d\_

**Definition:** fvar.hpp:16

lbeta.hpp

stan::agrad::fvar::val\_

T val\_

**Definition:** fvar.hpp:15


---

[ Stan Home Page ]

*© 2011–2014,
Stan Development Team.*
